# Two dimensional electrophysiological characterization of human pluripotent stem cell-derived cardiomyocyte system

**DOI:** 10.1038/srep43210

**Published:** 2017-03-07

**Authors:** Huanqi Zhu, Kelsey S. Scharnhorst, Adam Z. Stieg, James K. Gimzewski, Itsunari Minami, Norio Nakatsuji, Haruko Nakano, Atsushi Nakano

**Affiliations:** 1 Department of Chemistry and Biochemistry, University of California, Los Angeles, USA; 2 California NanoSystems Institute, University of California, Los Angeles, USA; 3 WPI Center for Materials Nanoarchitectonics (MANA), National Institute for Materials Science (NIMS), Japan; 4 Jonsson Comprehensive Cancer Center, University of California, Los Angeles, USA; 5 Institute for Integrated Cell-Material Sciences (WPI-iCeMS), Kyoto University, Japan; 6 Institute for Frontier Medical Sciences, Kyoto University, Japan; 7 Department of Molecular Cell and Developmental Biology, University of California, Los Angeles, USA; 8 Eli and Edythe Broad Center of Regenerative Medicine and Stem Cell Research, University of California, Los Angeles, USA; 9 Molecular Biology Institute, University of California, Los Angeles, USA

## Abstract

Stem cell-derived cardiomyocytes provide a promising tool for human developmental biology, regenerative therapies, disease modeling, and drug discovery. As human pluripotent stem cell-derived cardiomyocytes remain functionally fetal-type, close monitoring of electrophysiological maturation is critical for their further application to biology and translation. However, to date, electrophysiological analyses of stem cell-derived cardiomyocytes has largely been limited by biologically undefined factors including 3D nature of embryoid body, sera from animals, and the feeder cells isolated from mouse. Large variability in the aforementioned systems leads to uncontrollable and irreproducible results, making conclusive studies difficult. In this report, a chemically-defined differentiation regimen and a monolayer cell culture technique was combined with multielectrode arrays for accurate, real-time, and flexible measurement of electrophysiological parameters in translation-ready human cardiomyocytes. Consistent with their natural counterpart, amplitude and dV/dt_max_ of field potential progressively increased during the course of maturation. Monolayer culture allowed for the identification of pacemaking cells using the multielectrode array platform and thereby the estimation of conduction velocity, which gradually increased during the differentiation of cardiomyocytes. Thus, the electrophysiological maturation of the human pluripotent stem cell-derived cardiomyocytes in our system recapitulates *in vivo* development. This system provides a versatile biological tool to analyze human heart development, disease mechanisms, and the efficacy/toxicity of chemicals.

The potential of human pluripotent stem cells (hPSC) to self-renew indefinitely and differentiate into virtually any cell type makes them a valuable cell source for human developmental biology, cell-based regenerative therapy, disease modeling, and drug discovery/assessment^1^,^2^,^3^,^4^,^5^,^6^,^7^. As the human heart is the least regenerative of tissues, cardiomyocytes derived from human embryonic stem cell/induced pluripotent stem cells (hESC/iPSC-CMs) provide a particularly powerful biological tool^8^,^9^,^10^,^11^,^12^,^13^. Differentiation protocols have evolved over the years to allow for large-scale induction of human cardiomyocytes, and efforts have been made to induce further maturation of ESC/iPSC-CMs *in vitro*, with tissue engineering approaches showing promising results^14^,^15^,^16^,^17^. However, the maturity of *in vitro* hESC/iPSC-CMs still remains fetal-type with limited electromechanical properties. Unlike postnatal cardiomyocytes, hESC/iPSC-CMs are proliferative^14^,^18^,^19^,^20^, but with immature sarcomere structure^18^,^19^,^20^ and Ca^2+^ handling properties^14^,^21^,^22^,^23^,^24^. Sarcolemmal-dependency of calcium kinetics, negative force-frequency relation^21^ and high maximum diastolic potential demonstrate the functional immaturity of hESC/iPSC-CMs. Indeed, contractile forces generated by hESC/iPSC-CMs are estimated to be less than 0.5–5% of cardiomyocytes isolated from the neonatal heart^25^,^26^. Application of external biophysical cues including mechanical force^14^, electrical stimulation^17^, and matrix stiffness^13^,^27^ are promising approaches to induce the maturation of hESC/iPSC-CMs. However, these approaches have yet to achieve sufficient functionality to replace the damaged cardiomyocytes in the diseased heart.

Following the induction of the cardiac gene program during early embryogenesis, cardiomyocytes undergo a remarkable maturation process to develop into structurally and functionally competent cardiomyocytes during fetal stages, characterized by the assembly of contractile proteins into myofibrillar structure, expression of ion channels and gap junctions at the right location, organization of mitochondria and SR along the myofibrils, etc. The study of late-stage cardiogenesis is translationally relevant, as most of the events leading to congenital heart anomalies occur at later stages, and cell therapy requires functional cardiomyocytes with strong contractile force. Although great progress has been made in studies of specification and multilineage differentiation of cardiac progenitors, our understanding of the cardiac maturation process remains primitive^28^. While the differentiation level of early cardiac progenitors is well defined by marker gene expression, the maturity of cardiomyocytes at late developmental stages is relatively less reflected in the gene expression pattern^29^,^30^,^31^,^32^. Given that the biophysical cues are not only the results but also the essential drivers of the cardiac maturation^13^,^27^,^33^,^34^,^35^, the electrophysiological properties of hESC/iPSC-CMs are critical parameters to monitor.

Microelectrode arrays (MEAs) provide a highly sensitive, non-invasive method to study the electrophysiology of cardiomyocytes with spatiotemporal resolution. However, to date, the application of MEA to human cardiomyocytes has largely been limited by biologically undefined factors including 3D nature of embryoid body, sera from animals, and feeder cells isolated from mouse^5^. In this paper, using two-dimensional monolayer cultures of hESC-CMs with media free of animal products, we present a hybrid method for real-time measurement of electrophysiological dynamics of human cardiogenesis that is compatible with existing MEA technologies. Combination of hESC/iPSC-CM monolayer culture and the MEA system enables accurate, real-time, and flexible measurement of electrophysiological characteristics, thereby providing a versatile biological tool to analyze human heart development, understand disease mechanism, and assess the efficacy and toxicity of drugs.

## Results

### Molecular and cellular characterization of hESC-CMs

**Figure 1:**
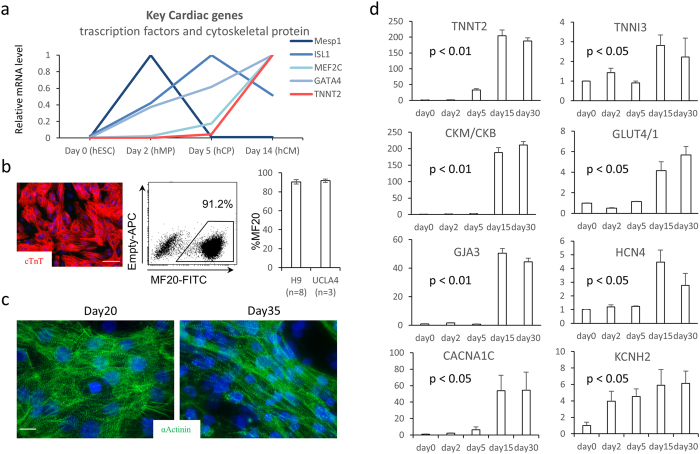
(**a**) mRNA relative expression over the time course of hESC differentiation toward cardiac lineages. The value is standardized to the peak value of the time points for each gene. (**b**) Immunofluorescent staining for α-actinin in red showing over 90% are positive for α-actinin (scale bar = 100 um) (**c**) Morphological changes in hESC-CMs over the time course. Representative optical images of hESC-CMs on MEA on Day 20 and Day 35 (fluorescent staining: α-Actinin in green, nucleus in blue, scale bar = 10 um). (**d**) mRNA relative expression by qPCR standardized to the expression of day0 over the time course of cardiac differentiation/maturation.

H9 and UCLA4 hESCs were grown and differentiated as previously described^12^ and plated as a monolayer. To define the differentiation stages, marker gene expression was serially profiled (Fig. 1a). mRNA quantification suggests that mesodermal markers (Mesp1, Bry) were highest at day 2 (mesodermal precursor stage; MP stage). Cardiac progenitor marker, ISL1, reached its peak at day 5 (cardiac progenitor stage; CP stage). Spontaneous contraction starts at around day 7. By day 14, major cardiac structural proteins, ion channels, and gap junctions became strongly expressed (immature cardiomyocyte stage; CM stage). At this stage, ~90% of the cells were MF20^+^ cardiomyocytes with typical sarcomeric structures (Fig. 1b). hESC-CMs underwent further maturation after day 14 and became elongated and oriented perpendicular to the lateral registration of sarcomeres by day 30 (Fig. 1c). α-actinin staining suggests that the Z-band becomes more aligned at later stages (Fig. 1c).

To examine the maturation level of late-stage hESC-CMs, the expression of marker genes at day 14 and day 28 were examined. Cardiac maturation indices, such as the subtype ratio of enzymes (CK-M/B) and metabolic indicators (GLUT4/1), progressively increased (Fig. 1d). However, the expression level of most contractile proteins (TNNT2, TNNI3), gap junction genes (GJA 3), or ion channel genes (HCN4) were not significantly upregulated in mRNA at day 28 compared to day 14 (Fig. 1d). Therefore, the mRNA expression profile was highly distinctive at early differentiation stages (days 2–14) but became less definitive at late maturation stages (days 14–28).

### Validation of MEA culture

**Figure 2:**
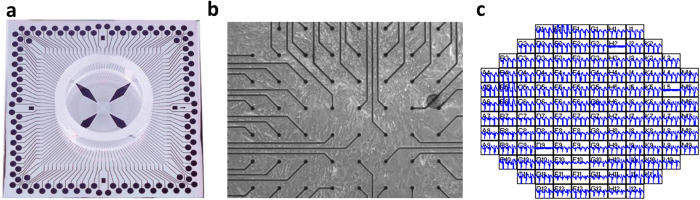
(**a**) Microelectrode arrays (MEA) with 120 integrated TiN electrodes (30 μm diameter, 200 μm inter-electrode spacing) were used to culture the 2D hESC-CMs. (**b**) Representative optical image of hESC-CMs on top of MEA. (**c**) Field potential signals were detected in electrodes which are in touch with the hESC-CMs.

**Figure 3:**
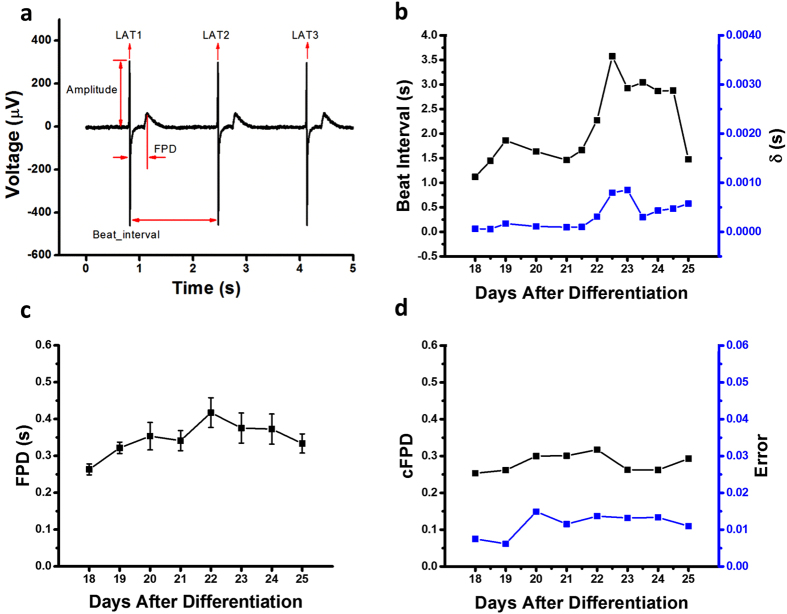
(**a**) Field potential features such as beat interval, field potential duration, amplitude, and local activation time (LAT) were extracted out of the signal sequences. On average, 100 out of 120 channels were selected to calculate the beat interval after the peak detection and experiments were performed in triplicate. (**b**) The beat interval was stable up to day 28–30 with a beat interval around 2 s. Starting from day 28, the beat interval of hESC-CMs became unstable and irregular. The standard deviation (δ) of beat interval agreed with the beat interval trend. (**c**) The field potential duration (FPD) ranged from 0.2 s to 0.5 s and was relatively stable throughout the differentiation process. (**d**) The corrected FPD ( = FPD/[beat interval/1000]^1/3^; Fridericia’s formula) was also relatively stable ranging from 0.2 to 0.3.

Taking advantage of the monolayer differentiation regimen, hESC-CMs were cultured on an MEA platform that enables real-time (up to 40,000 samples/sec), simultaneous acquisition of 120-channels of spatially distributed electrical data from individual microelectrodes embedded in a cell culture device (Fig. 2a–c). Inadequate contact between cultured cells and the measurement electrodes resulted in signal amplitudes less than a pre-defined signal-to-noise ratio (4), making them indistinguishable from the background noise of the measurement system. Electrode channels in firm contact with the underlying cell monolayer acquired clearly distinguishable local field potential signals (Fig. 3a) that enabled examination of hESC-CMs maturation at the electrophysiological level by monitoring their steady and consistent beat signatures over 10 days when cultured on an MEA plate. Regular beat intervals ranging from 2–4 s with standard deviations below 0.0014 s (Fig. 3b) and field potential durations (FPDs) of approximately 0.3–0.4 s were observed throughout the differentiation (Fig. 3c). Corrected FPD was also relatively stable (Fig. 3d).

**Figure 4:**
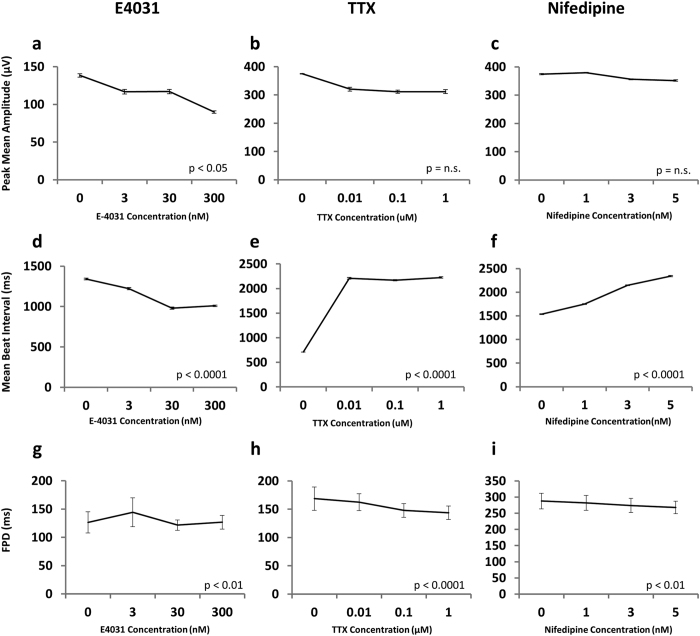
Peak mean amplitude (**a**–**c**) and mean beat interval (**d**–**f**) of field potential of monolayer cardiomyocytes cultured in the presence of (**a**,**d**) E4031 (K^+^ channel blocker), (**b**,**e**) TTX (tetrodotoxin, Na^+^ channel blocker) and (**c**,**f**) Nifedipine (Ca^2+^ channel blocker). p-values are calculated by one-way ANOVA. Data are representative of at least 2 biological replicates.

Monitoring the field potential of hESC/iPSC-CMs following chemical compound administration has been reported and expected as a novel drug screening tool for cardiotoxicity. To validate the assay system, response to pharmacological interventions were examined by administration of canonical ion channel blockers E4031, TTX (tetrodotoxin), and Nifedipine (Fig. 4). Consistent with previous reports^6^, E4031, a K^+^ channel blocker, reduced the field potential amplitude and the beat interval in a concentration-dependent manner but had no impact on FPD (Fig. 4a,d,g). TTX, a selective Na^+^ channel blocker, induced an increase in the beat interval even at a remarkably low concentration with a concurrent trend of reduction in the amplitude and FPD (Fig. 4b,e,h). Nifedipine, a Ca^2+^ channel blocker with negative inotropic and chronotropic effects, showed a mild trend of reduction in the peak amplitude of field potential, a significant increase in the beat interval, and decrease in FPD (Fig. 4c,f,i), consistent with well-known clinical observations. Previous reports using hESCs showed a decrease in beat interval with Nifedipine^36^. These inconsistencies may be attributed to the specific details of differentiation regimens, relative condition of the cells, or inter-cell line difference. The observed reduction in peak amplitude in response to E4031 was deemed statistically significant based on a p value of less than 0.05 in an analysis of variance (ANOVA). Trends observed for the reduction in peak amplitudes for TTX and Nifedipine were found to be statistically insignificant. Trends, both increases and decreases, in observed beat intervals for all three ion channel blockers were also statistically significant according to their representative p-values. These results suggest that our system facilitates an accurate and real-time measurement of the electrophysiological activity of hESC-CMs^6^,^36^.

### Identification of pacemaking cells in the monolayer cardiac sheet

**Figure 5:**
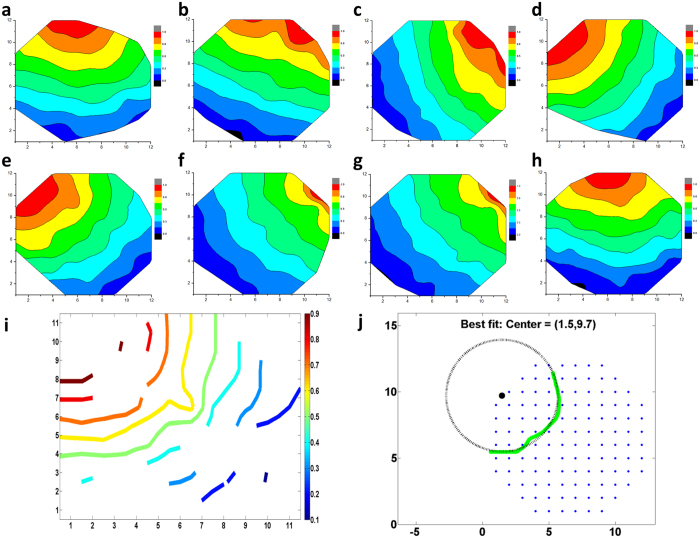
Field potential propagation, pacemaking and conduction velocity of the 2D hESC-CMs. (**a**–**h**) shows a representative activation map from day 19, 20, 21, 22, 25, 26, 28, 29, respectively. The activation map was consistent over the 20-minute recording window showing a stable pace making source, but the propagation map changed over days. (**i**) On day 23, a contour graph of the propagation wave was generated from the activation map. (**j**) The center of the propagation wavelet was identified by fitting the wavelet with a circle. The origin of the circle represented the location of the pacemaking cells. The pacemaking cells were located at the edge of the MEA.

The combination of a confluent layer of hESC-CM and the large microelectrode array allowed for not only temporal, but also for spatial analysis of the electrical activity. The field potential propagation was characterized using the phase difference of each channel covered by the confluent layer of hESC-CM. Figure 5 shows representative field potential propagation waves at varying cell age from day 18 to 25. The propagating wave generates circular wave fronts. Analysis over a 20-minute recording window showed a consistent propagation map, indicating that the pacemaking source was stable (Fig. 5a–d). However, the propagation map changed over days, indicating that the pacemaking source was not completely fixed over a long period (Fig. 5e–h,i).

The electrical activity of hESC-CMs was initialized by a small group of pacemaking cells and propagated to the surrounding cardiomyocytes via gap junctions. Assuming that the propagation waves originated from a point source, the contour of the activation map was approximated by a circle. Although the position of pacemaking cells may fall outside of the electrode array in the MEA platform (see Fig. 2), the location of pacemaking cells can be predicted using the radius of the curvature of the field potential. For example, the coordinates of the pacemaking cells shown in Fig. 5j were (1.5, 9.7), at the edge of the MEA electrodes.

### Characterization of cardiac maturity and conduction velocity

**Figure 6:**
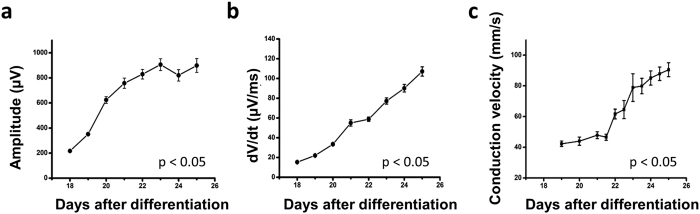
(**a**) The field potential amplitude rapidly increased until day 23 and declined at day 27. The turning point agreed with that of the beat interval. (**b**) The field potential amplitude upstroke speed increased from 15.3 μV/ms at day 18 to 122.9 μV/ms at day 26 and declined at day 27. (**c**) Conduction velocity was calculated at each day. There was an increment of conduction velocity as the development of hESC-CMs. Results from two samples were consistent.

The capacity to monitor spatiotemporally resolved local field potentials in real-time provides a readout for maturation of hESC-CMs during differentiation. Consistent with a previous study^30^, the mean field potential amplitude increased rapidly (4–5 fold) up to day 26 (Fig. 6a). Furthermore, the maximal field potential upstroke (dV/dt max), indicative of the sodium channel activity, became progressively larger as the hESC-CMs maturate (Fig. 6b). During natural embryogenesis, cardiac conduction velocity increases as the heart maturates, with neonatal and adult cardiomyocytes showing ~0.3 m/s and ~1 m/s, respectively. To examine whether conduction velocity increases during the maturation of hESC-CMs in this system, the conduction speed was calculated by analyzing the signal traveling distance and time lag. Analysis performed in both vertical and diagonal directions with respect to the pseudo pacemaking region (Fig. 6c) reveal a conduction speed at day 18 of approximately 35 mm/s. As the hESC-CMs maturated over subsequent days, conduction velocities increased up to a maximum of 120mm/s at day 28, a value indicative of functional immaturity relative to neonatal cardiomyocytes. Field potential amplitude, upstroke and conduction velocity were determined to be statistically significant based on p-values < 0.05 determined via ANOVA. In contrast to commonly utilized molecular signatures, electrophysiological parameters showed a clear progression of cardiac maturity during the differentiation stages between days 14 to 26. Thus, the combination of the monolayer culture platform and MEA facilitates comprehensive monitoring of the electrophysiological maturation process of hESC-CMs.

## Discussion

The integration of a chemically-defined differentiation regimen with a monolayer culture of human pluripotent stem cell-derived cardiomyocytes and electrophysiological measurement on an MEA platform provides a direct method to examine the spatiotemporal dynamics of the electromechanical maturation in human cardiomyocytes. Stem cell derived cardiomyocytes provide a powerful tool for human developmental biology, regenerative therapies, disease modeling, and drug discovery. However, the utility of MEA for the hESC/iPSC-CMs has been largely limited to disease modeling and drug assessment^7^,^37^,^38^,^39^,^40^,^41^,^42^,^43^,^44^. The monolayer culture method described here overcomes technical problems associated with the utilization of conventional 3D embryoid bodies, namely large inter-experiment variability due to inconsistency in their size, quality, and attachment to MEA surface. In addition, the use of animal sera and feeder cells often introduce additional variables to differentiation efficiency and functionality. Our chemically-defined regimen serves to further minimize experiment-to-experiment variability^12^. In combination, our system enables the monitoring of electric signals over weeks from cells in direct contact with electrodes, identification of pacemaking cells, and performance of computational analyses of localized voltage-type signal and position-dependent properties of electrical activity.

Utilization of this system has enabled a direct characterization of electrophysiological maturation in hESC-CMs. During the natural embryonic cardiogenesis, action potential amplitude, action potential upstroke, conduction velocity, and heart rate gradually increase as the fetal cardiomyocytes maturate. *In vitro* differentiation of pluripotent stem cells recapitulates the natural developmental process. Our data suggest that hESC-CM differentiation shows the same trend of electrophysiological properties including field potential amplitude, dV/dt max, and conduction velocity. Even though our differentiation protocol is ‘directed’ toward the cardiac lineage, the differentiation of hESC-CMs follows the same developmental process at genetic and electrophysiological levels. Of note, the electrophysiological parameters show a progressive increase in the field potential amplitude, dV/dt, and conduction velocity (Fig. 6), while typical cardiac markers including TNNT2 and TNN13 do not show significant differences after day 14 (Fig. 1). Therefore, electrophysiological parameters appear to be more sensitive indicators for the assessment of cardiomyocyte maturity than genetic parameters. Although our monolayer hESC-CMs display robust beating, the electrophysiological parameters indicate that monolayer hESC-CMs are less differentiated than cardiomyocytes generated via 3D embryoid bodies. For example, previous reports show dV/dt max of 2 V/second or higher^45^,^46^. The relative immaturity of hESC-CMs may be in part because the monolayer condition is less physiological than the 3D environment. Possibly due to the immaturity or to the spatial resolution of electrode array, the wave fronts were not reorganized from circular to oval pattern as hESC-CMs differentiate. These limitations should be considered when applying the monolayer hESC-CM MEA system to analyses of human developmental biology, patient-specific disease mechanisms and drug discovery/assessment.

Looking ahead, the availability of high-throughput measurement platforms has proven to be highly advantageous in basic and translational research. High-throughput optical readouts using voltage dyes, calcium dyes, or their combinations have demonstrated the utility of hESC/iPSC-CMs as a model system for such methods^6^,^47^. While our current system configuration cannot be directly utilized in high-throughput analysis, there is no fundamental barrier that precludes the development of such capabilities. In fact, monolayer culture facilitates the basic molecular and cell biology techniques including lipofection, viral infection, and microscopic observation in combination with the electrophysiological analyses described here. Further, when considering the reproducibility and the versatility of the MEA platform coupled with ~90% cardiac efficiency in our monolayer culture, the development of high-throughput electrophysiological readouts represents a promising research direction. The accurate, real-time, and flexible method demonstrated in this study sets a new standard for electrophysiological analyses of hESC/iPSC-CMs with broad applicability in stem cell biology.

## Methods

### Microelectrode array measurements

hESC-CMs were maintained and differentiated as previously described^12^. At 16 days of age hESC-CMs were plated on uncoated, microelectrode arrays (MEAs) containing 120 integrated TiN electrodes (30 μm diameter, 200 μm interelectrode spacing). The MEAs were placed in an incubator with a temperature of 37 °C and 5% CO_2_. Two days were given to ensure the cardiomyocytes were well attached to the MEA. Recording commenced at a cell age of 18 days. Local field potentials at each electrode were collected over a period of 20 minutes, twice a day, and 3 weeks in total with a sampling rate of 1 KHz using the MEA2100-HS120 system (Multichannel systems, Reutlingen, Germany). The chemical compounds used herein were purchased from Tocris (E4031), Fisher Scientific (TTX) and Sigma-Aldrich (Nifedipine).

### Peak detection

Determining the amplitude, frequency, and conduction velocity of cardiomyocyte activity required reliable assignment of peaks in the local field potential signal for all 120 channels. The Matlab function ‘findpeaks’ was used with tunable parameters ‘minpeakheight’ and ‘minpeakdistance’ to reliably determine signal peaks. Setting the ‘minpeakheight’ as 16 μV defined a minimum signal-to-noise ratio of 4 considering the noise level in the MEA system was ± 8 μV. The ‘minpeakdistance’ was set as 1000 ms, which was based on the experimental observation that cardiomyocytes were beating at a frequency smaller than 1 Hz.

### Beat interval

The beat interval was measured as the peak-to-peak time difference from the beat amplitude assignments for each channel. After implementing peak detection as described above, the beat interval and standard deviation were collected for all channels. Unsynchronized and/or significantly noisy electrodes excluded from analysis were those with standard deviations that fell outside of 10% of the median standard deviation from all channels. The mean of the beat interval ± STD was calculated from all channels within this threshold.

### Analyses of the field potential propagation

Time differences in the rhythmic local field potential collected from 120 electrodes were quantified by dividing the complete data set for each day into a sequence of segments (typically 30 s, each). Peak detection was used to identify active channels. Cross correlation of the acquired signals from each active channel with respect to a pre-chosen reference electrode provided the time lag of each electrode with respect to the reference electrode. The time lags were then normalized between 1 and 0, representing the pace leader and last follower, respectively.

### Identification of pacemaking cells

The pacemaking cells sometimes fall outside the region of the MEA electrode area. Assuming the whole cardiomyocyte culture had only one cluster of pacemaking cells and the pace-generating signal propagated like a wave, the position of the pacemaking cells can be extrapolated based on the partial propagation waveform that was detected in the MEA electrode area. Compared to the localized pacemaking cells, a contour plot of propagation time at each channel within the MEA electrode area was shown. Each archer-shape line had the same propagation time. A circle was fitted to the archer-shape line and the origin of the circle represented the location of the pacemaking cells. A Matlab circle fit module developed by Izhak Bucher was adopted in the above process.

### Conduction speed analysis

Conduction speed was defined as the velocity of the field potential propagation across the MEA surface. The time for each electrical beat was found through the peak detection analysis described previously. The time lag and distance of each channel with respect to the pacemaking cells (earliest beat identified from the 120 channels) was calculated. The conduction speed at each channel was therefore calculated. Conduction speed in both the vertical and diagonal direction was shown in Fig. 6c. To calculate the average conduction speed for each recording period, the conduction speed was averaged in both 120 channels and the 20-minute recording period. Results from a cell age of 18 days to 25 days were shown in Fig. 6.

## Additional Information

**How to cite this article**: Zhu, H. *et al*. Two dimensional electrophysiological characterization of human pluripotent stem cell-derived cardiomyocyte system. *Sci. Rep.* **7**, 43210; doi: 10.1038/srep43210 (2017).

**Publisher's note:** Springer Nature remains neutral with regard to jurisdictional claims in published maps and institutional affiliations.
